# Mad Honey Poisoning in Nepal: A Case Report

**DOI:** 10.1002/ccr3.70069

**Published:** 2025-01-06

**Authors:** Md Fahad Hossain, Manish Kharel

**Affiliations:** ^1^ Ministry of Health and Family Welfare Kuliarchar Bangladesh; ^2^ Kathmandu Medical College Kathmandu Nepal

**Keywords:** cardiac arrhythmia, grayanotoxin, mad honey, Nepal, poisoning, toxin

## Abstract

Widespread vagal activation following honey consumption is a sign of mad honey poisoning. Early initiation and appropriate treatment can prevent fatal outcomes.

## 1 Introduction

Modern science has shown the value of honey as a medicine. It possesses extensive antibacterial, antiviral, and antifungal properties, through several mechanisms, including low pH and enzyme activity. Honey both inhibits and eliminates microorganisms [1]. But sometimes, wild honey (sometimes known as “mad honey”) produced by bees from specific rhododendron species might result in honey poisoning. Because it is tainted with grayanotoxin, mad honey differs from commercial or marketed honey and is referred to as intoxicating [2]. Many parts of the world, including Turkey, Japan, Nepal, North America, and Brazil, have plants that are high in the grayanotoxin that causes honey intoxication [3, 4]. Certain parts of the world generate mad honey; they include Nepal (5%), and the Black Sea region of Turkey (91% of poisoning cases in one investigation). Mad honey produced in Turkey was responsible for the bulk of poisoning cases (91.44%), followed by Nepal (4.67%) and Korea (1.56%) [5]. The Black Sea region is home to the majority of documented cases of mad honey disease [6]. However, occurrences in other countries may arise from the transportation and export of locally produced honey from Turkey or elsewhere, as well as from the geographic dispersal of the Turkish people. Certain examples, for instance, have been reported from Korea [7, 8, 9], Germany [10, 11], Austria [12, 13], Switzerland [14], and other regions of Turkey [15]. Significant volumes of poisonous honey, which has been outlawed since 2005, were imported by the latter nation [7]. Furthermore, the incidence of grayanotoxin intoxication by honey produced outside of Turkey is highlighted by a few old and recent cases of local wild honey poisoning from Nepal [16, 17, 18] as well as Reunion Island [19]. Widespread vagal activation following honey consumption is a sign of mad honey poisoning. Early initiation and appropriate treatment can prevent fatal outcomes. This is a rare life‐threatening condition for which there is not appropriate investigation method present in Nepal. So, careful history taking and physical sign will guide physician to manage this condition appropriately.

## 2 Case History

A 65‐year‐old male came to the hospital with complaints of nausea, vomiting, and dizziness 25 min after the ingestion of 30 mL of honey. The patient has no additional noteworthy past medical history, similar previous attacks, or hypotension. Neurologically the patient's altered mental state was evident upon evaluation. On cardiovascular examination, her blood pressure was 60/40 mm hg, pulse rate was 40 beats per minute, and regular. Other physical examination findings include a temperature of 98.7 °F (37.05°C), respiratory rate of 18/min, and oxygen saturation of 98% at room air.

## 3 Investigations, Diagnosis, and Treatment

**FIGURE 1:**
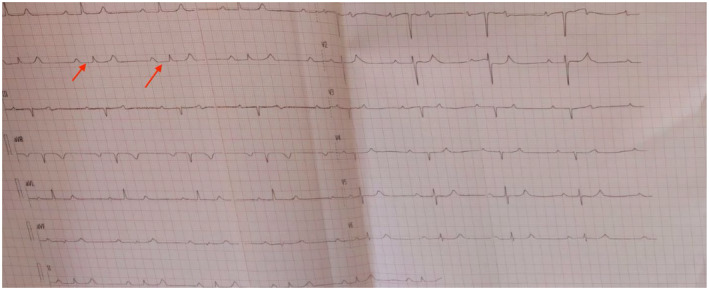
ECG of the patient of honey bee poisoning during admission. First‐degree heart block with heart rate:38 bpm.

Immediate resuscitation was done with 1000 mL intravenous normal saline bolus, 20 mcg/kg/min IV dopamine to elevate blood pressure, and 1 mg IV atropine bolus single dose to elevate heart rate 0.12 lead ECG revealed patient has first‐degree heart block with a heart rate (HR) of 38 bpm Figure 1. A blood investigation was done, which is shown in the following Table 1.

**TABLE 1:** Blood report of the patient with mad honey poisoning without much abnormality.

Blood test	
Hemoglobin (12–16 g/dL)	12.33
Red blood cells (60–200 mg/dL)	108
Serum sodium (135–145 mmol/L)	140
Serum potassium (3.5–4.5 mmol/L)	4
Blood urea nitrogen/serum creatinine	26/1.1
Thyroid‐stimulating hormone (0.4–4.2 mIU/L)	2.5

After ruling out all other diagnoses based on acute presentation, absent medical illness, laboratory findings, and taking into account the patient's history of consuming honey, specific geographical location (far western part of Nepal), and heart block symptoms, the diagnosis of honeybee poisoning was made. The patient was then transferred to the intensive care unit (ICU), where she was given 2000‐mL intravenous (IV) of normal saline, 100 mg of hydrocortisone three times per day, 20 mcg/kg/min IV dopamine to elevate blood pressure, and 1 mg IV atropine to elevate heart rate. His blood pressure and mental status did not improve until 24 h after admission and remained at 80/60.

## 4 Outcome and Follow‐Up

**FIGURE 2:**
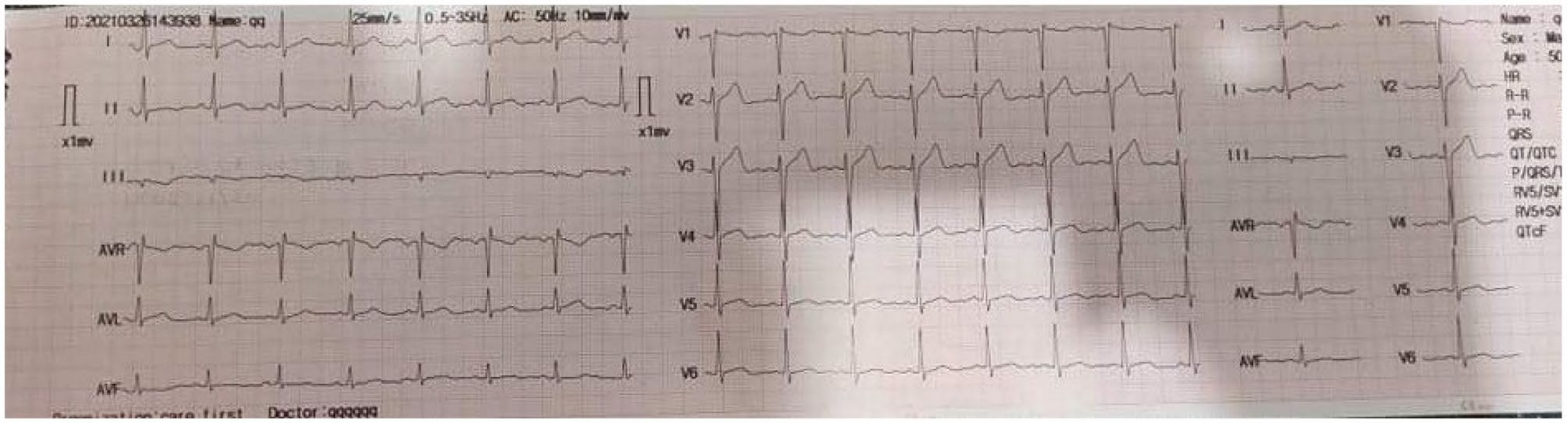
ECG of the mad honey poisoning patient during discharge, Normal sinus rhythm.

After 60 h of admission, blood pressure started to increase to 100/60 mmHg, and her pulse rate to 58 beats per minute after receiving 3000 mL of normal saline and dopamine. The mental status of the patient was also improved. After that, the dopamine and IV fluid were stopped. After 90 h, his blood pressure was gradually stabilized to 120/80 mmHg with a heart rate of 70 beats per minute. Figure 2 shows the normal ECG finding after stabilization. After being admitted to the intensive care unit for 120 h, the patient was shifted to the general wards and underwent cardiac monitoring there until being discharged.

## 5 Discussion

Consuming wild honey produced by bees from rhododendron species contain the deadly compound known as grayanotoxin, which causes honey poisoning. Grayanotoxin's capacity to block voltage‐gated sodium channels found in neuronal cell membranes is what gives it its poisonous properties [6].

Grayanotoxin I is one of the 18 types of grayanotoxins and is the cause of honey poisoning, which occurs in plants belonging to Ericaceae plants [20, 21]. Bees that gather nectar from these plants cannot get rid of the toxin known as grayanotoxin, which is a form of neurotoxin. Therefore, it becomes absorbed into the honey. Thin‐layer chromatography and paper electrophoresis are adopted as standard procedures for grayanotoxin detection [2]. Consumption of about 15–30 g of mad honey leads to intoxication, and symptoms appear (2.8 ± 1.8 h) after ingestion [1, 16].

Although there is a linear relationship between toxic effects and the quantity of mad honey consumed, the distribution of grayanotoxin inside honey is not uniform, which causes varying amounts of intoxication in various patients. When eaten, the grayanotoxin binds to voltage‐gated sodium channels, preventing them from inactivating, resulting in the activation of Bezold‐Jarisch Reflex (BJR), which inhibits heart function. The heart rate slows (bradycardia), blood pressure remains consistently low, and peripheral vasodilation results from the central vasomotor centers being inhibited. Through stimulation of the unmyelinated afferent cardiac branches of the vagus nerve, this is accompanied by a decrease in sympathetic output and peripheral vascular resistance. For this reason, excess cholinergic symptoms are similar to those of mad honey poisoning [20].

Blood and urine samples can be tested for grayanotoxin levels. However, none of the numerous hospitals in Nepal, including our own, have the capability to measure toxic levels. We were unable to determine the amounts of grayanotoxin in our patients. In our case, mad honey intoxication is diagnosed based on clinical observations, and it is often suspected in patients who experience typical signs and symptoms after consuming honey but have no prior history of cardiac problems. In regions where mad honey poisoning is prevalent, the patient's history of honey consumption before the onset of symptoms is usually sufficient to make a diagnosis. However, currently, there is no routine test available to measure the level of grayanotoxin in a patient's blood [2]. The symptoms of mad honey poisoning include a burning sensation in the throat and upper abdomen, as well as vomiting, diplopia (double vision), blurred vision, dilated pupils, sluggish reaction to light, palpitations, hypotension, blackouts, loss of consciousness, hallucination, deep gasping, and apnea [6]. Reversible neurological and behavioral symptoms can also arise in patients, even though cardiovascular symptoms are the most common like in our case [22]. A number of cases of mad honey poisoning also result in hypothermia [21]. Older adults (those over 65) experience poisoning at an earlier age, with symptoms lasting longer [23]. The majority of patients experience myocardial infarction, bradycardia, and heart blocks of varying degrees [15, 24, 25]. The most common findings in ECG were: sinus bradycardia (79.58%), complete atrioventricular block (45.83%), atrioventricular block (30.91%), ST‐segment elevation (22.63%), and nodal rhythm (11.27%) [26].

Saline infusion is used to treat dizziness and mild hypotension, whereas atropine is chosen in the event of severe hypotension and bradycardia. Forty‐seven cases of mad honey poisoning presented to the 3 health institutions in 2007 were investigated in which 0.5 to 2 mg of atropine was given to all patients [27]. Atropine improves heart rate, thereby improving the BP of the patient. Transvenous pacing and epinephrine or dopamine infusion are indicated if saline infusion and atropine are insufficient. Three patients have used a transvenous temporary pacemaker as of this writing due to asystole and total heart block [2, 16]. Most cases of mad honey poisoning are rarely fatal (8 out of 31 patients (25.8%) in Lansing County, Southeast China) managed symptomatically, and usually resolve within 24 h [16]. However, in our situation, the patient's condition did not stabilize completely for another 90 h. Honey poisoning is uncommon and only happens in specific geographic places. So, physicians should be cautious about the condition. To avoid mad honey poisoning and the difficulties associated with poisoning, it's also critical to raise public awareness. Patients' lives can be saved by appropriately diagnosing and managing them. Further research is required to better understand the illness. Medicinal plants and silver nanoparticles studies are showing promising results in mad honey poisoning and demand continued studies [28].

In conclusion, mad honey containing grayanotoxin is responsible for the potential effect of its poisoning. There is no test available to check the level of grayanotoxin for diagnosis, which is done on the basis of history, clinical features, and highly suspicious geographical location. Although the symptom resolves within 24 h, some patients need a longer time for recovery. Fewer new research is going on, which can open new doors for the management of mad honey poisoning in the future.

## Author Contributions

**Md Fahad Hossain:** conceptualization, software, writing – original draft, writing – review and editing. **Manish Kharel:** conceptualization, resources, writing – original draft.

## Consent

A written informed consent was obtained from patient to publish this report in accordance with the journal's patient consent policy.

## Conflicts of Interest

The authors declare no conflicts of interest.

## Data Availability

The data that support the findings of this study are available on request from the corresponding author. The data are not publicly available due to privacy or ethical restrictions.
